# Baseline PSMA tumor volume as a prognostic marker in radical radiotherapy for prostate cancer: a propensity score-weighted retrospective analysis

**DOI:** 10.1007/s12149-025-02118-4

**Published:** 2025-10-10

**Authors:** Francesco Lanfranchi, Liliana Belgioia, Daniele Vita, Jacopo Passoni, Sara Mastrogiovanni, Alessandra Catanoso, Luca Sofia, Valentina Pau, Stefano Raffa, Silvia Chiola, Maria Isabella Donegani, Roberta Piva, Mattia Riondato, Michela Marcenaro, Giorgia Timon, Cecilia Marini, Salvina Barra, Gianmario Sambuceti, Matteo Bauckneht

**Affiliations:** 1 Department of Experimental Medicine (DIMES), University of Genova, Genoa, Italy; 2 Department of Health Sciences (DISSAL), University of Genova, 16132 Genoa, Italy; 3 IRCCS Ospedale Policlinico San Martino, Genoa, Italy; 4 Institute of Bioimaging and Complex Biological Systems, National Research Council (CNR), Milan, Italy

**Keywords:** Prostate cancer, Prostate-specific membrane-antigen, Positron emission tomography, PSMA-tumor volume, Radiotherapy

## Abstract

**Objective:**

Next-generation imaging with prostate-specific membrane-antigen Positron Emission Tomography/Computed Tomography (PSMA PET/CT) has emerged as an imaging modality offering high diagnostic accuracy and prognostic biomarkers in the primary staging of prostate cancer (PCa). Among these, PSMA-positive tumor volume (PSMA-TV) may carry prognostic significance but has been poorly investigated in patients receiving radical-intent radiotherapy (RT).

**Methods:**

Patients with biopsy-proven unfavorable intermediate-to-high-risk PCa staged as non-metastatic (T1-4 N0-1 M0) at [^68^ Ga]PSMA-11 or [^18^F]PSMA-1007 PET/CT before definitive RT plus androgen deprivation therapy (ADT) at our Institution (2019–2024) were retrospectively recruited. Following RECIP criteria, semi-quantitative PET parameters extracted were: maximum and mean standardized uptake value (SUVmax and SUVmean), PSMA-TV, and total lesion PSMA uptake (PSMA-TL) [PSMA-TV*SUVmean]). We assessed the association between PET-derived semi-quantitative parameters and clinical outcomes, including time to treatment failure (TTF) and PSA response. Inverse probability of treatment weighting (IPTW) was adopted to address confounders, namely, initial PSA, ISUP score, T stage, and N stage.

**Results:**

Among 145 patients recruited, median age was 76 years and median initial PSA 8.9 ng/mL. Most patients had ISUP grade ≥ 3 (39.3%), and 28.3% presented with nodal involvement at staging. Concurrent androgen deprivation therapy was administered in all patients, and the most common duration was 12–24 months (60.7%). The median follow-up was 20.5 months. While unadjusted analyses showed no significant association between PET parameters and treatment outcomes, IPTW-adjusted survival analysis revealed that high PSMA-TV was significantly associated with shorter TTF (*p* < 0.05). Other PET-derived metrics were not predictive of outcomes.

**Conclusion:**

Our findings highlight PSMA-TV as an independent predictor of treatment failure following definitive RT in PCa, supporting its potential role as a risk-stratifying biomarker, paving the way for individualized therapeutic strategies. Prospective validation is warranted to confirm its clinical utility and guide future radiotherapy personalization.

**Supplementary Information:**

The online version contains supplementary material available at 10.1007/s12149-025-02118-4.

## Introduction

Radiation therapy (RT) with or without androgen deprivation therapy (ADT) is a standard approach for the radical treatment of patients with non-metastatic (M0) prostate cancer (PCa) [1]. However, the growing diffusion of next-generation imaging techniques has shown how the definition of M0 patients relies on the diagnostic modalities adopted. In particular, Prostate-Specific Membrane Antigen (PSMA) Positron Emission Tomography/Computed Tomography (PET/CT) showed a higher accuracy in the clinical definition of the stage of disease with respect to conventional imaging [2–4].

Beyond its superior diagnostic performance, PSMA PET/CT also offers quantitative imaging biomarkers that may carry prognostic significance. Recent multicenter studies have demonstrated that total PSMA-positive tumor volume (PSMA-TV), a measure of disease burden derived from PET/CT, is associated with overall survival in patients with PCa, including those undergoing initial staging [5, 6]. Nevertheless, these studies did not include stratified analyses based on the definitive treatment received, and data on the prognostic utility of PSMA PET/CT-derived parameters in patients undergoing radical-intent radiotherapy remain lacking.

In this context, our study aimed to investigate whether semi-quantitative parameters obtained from pre-treatment PSMA PET/CT could provide independent prognostic information in patients with newly diagnosed PCa treated with radical-intent RT. By evaluating the association between PSMA-TV and time to treatment failure, we sought to explore its potential role as a risk-stratifying biomarker to guide personalized therapeutic strategies.

## Materials and methods

### Study design and patient population

Patients with biopsy-proven unfavorable intermediate-to-high-risk PCa submitted to PSMA PET/CT at the most six months before definitive radiotherapy, with or without ADT, at IRCCS Ospedale Policlinico San Martino (Genoa, Italy) between 2019 and 2024 were retrospectively recruited. Inclusion criteria were: i) biopsy-proven new diagnosis of unfavorable intermediate-to-high-risk PCa; ii) staging with PSMA PET/CT with either [^68^ Ga]Ga-PSMA-11 or [^18^F]F-PSMA-1007 staging; iii) PSMA PET/CT clinical stage T1-4, N0-N2 (N +), M0; iv) radical treatment with radiation therapy with or without ADT. Patients with age < 18 years, PCa with non-adenocarcinoma histology, and metastatic disease (M1a, M1b, or M1c) at staging PSMA PET/CT were excluded.

The Local Ethics Committee (Regione Liguria Ethical Committee, registration number 5/2023–DB id 12,914, date of approval 19-Jun-2023) approved the study. All procedures were performed in accordance with the ethical standards of the institutional and/or national research committee and with the 1964 Helsinki Declaration and its later amendments or comparable ethical standards. Due to the study’s retrospective design, informed consent was waived from the Ethical Committee.

### PSMA PET/CT imaging acquisition

For study enrollment, patients submitted to PSMA PET/CT with either [^68^ Ga]Ga-PSMA-11 or [^18^F]F-PSMA-1007 were considered. [^68^ Ga]Ga-PSMA-11 and [^18^F]F-PSMA-1007 PET/CT scans were acquired about 60 and 90 min after the tracer administration, according to the current guidelines [7].

PSMA PET/CT hybrid scans were acquired using either Siemens Biograph MCT Flow (matrix size 200 × 200, PSF-TOF reconstruction with 3 iterations and 21 subsets) or GE Healthcare Omni Legend (matrix size 256 × 256, VUE Point HD iterative reconstruction with 4 iterations and 8 subsets).

### PSMA PET/CT image analysis

All PET/CT images were visually assessed and semiquantitatively analyzed by a nuclear medicine physician with more than 10 years of experience (MB) according to the current reporting guidelines [8]. For visual analysis, areas presenting a focal uptake higher than the background were reported as suspected of malignancy. Images were evaluated for the detection of the primary tumor, locoregional and extra-pelvic lymph nodes, and bone and visceral metastases. The issue of Unspecific Bone Uptakes (UBU), frequently occurring in [^18^F]F-PSMA-1007 scans [9], was addressed using the BUMP score [10], based on lesions’ SUVmax and mean Hounsfield unit at co-registered CT images. The following semi-quantitative PET parameters were extracted using the LIFEx software version 7.8.0 [11]: maximum and mean standardized uptake value (SUVmax and SUVmean, respectively) of the dominant intraprostatic lesion, total PSMA-positive tumor volume (PSMA-TV), and total lesion PSMA uptake (PSMA-TL, calculated as the product between PSMA-TV and SUVmean). Semiquantitative parameters were calculated with a variable SUVmax threshold based on the background uptake (liver for [^68^ Ga]Ga-PSMA-11 and spleen for [^18^F]F-PSMA-1007) as per RECIP criteria [12]. Semi-automatic analysis was visually verified by a nuclear medicine physician with high experience. To avoid the inclusion of prostate areas with paraphysiological uptake, only regions defined as PRIMARY score of at least 3 were segmented [13]. Supplementary Fig. 1 shows an example of prostate gland segmentation.

### Clinical and treatment data

The clinical variables collected for analysis included: initial PSA level (ng/mL), ISUP grade group, T stage, N stage, and concurrent ADT.

After imaging, all patients received RT in accordance with international clinical guidelines for the management of unfavorable intermediate-to-high-risk PCa. RT was delivered using advanced techniques, either volumetric modulated arc therapy (VMAT) with linear accelerator (LINAC) or helical intensity-modulated radiotherapy (IMRT) by helical tomotherapy. All patients underwent hypofractionated RT schedules, prescribed according to institutional protocols and under the supervision of a board-certified radiation oncologist with expertise in PCa management.

### Data analysis

Continuous and categorical characteristics are presented as median (interquartile range [IQR]) and as absolute frequency (percentage), respectively. The PET parameters were binarized according to median values to define “low” and “high” groups. The following endpoints were assessed: PSA response, defined as a reduction of PSA levels of at least 50% (PSA50) or 90% (PSA90) compared to pre-treatment values; PSA relapse, defined as a post-radiotherapy increase of ≥ 2 ng/mL above the nadir (Phoenix criteria); Time to Treatment Failure (TTF), defined as the interval from radiotherapy to either biochemical, radiological, clinical progression, initiation of a new treatment, or death. Time to event was calculated in months from the date of radiotherapy.

### Statistical analysis

The inverse probability of treatment weighting (IPTW) approach was used to address confounding. Binary logistic regression was used to compute propensity scores, with the following parameters as covariates: initial PSA, ISUP score, T stage, and N stage. Covariate balance before and after weighting was assessed by calculating standardized mean differences (SMDs) for each confounder included in the propensity score model. SMDs below 0.1 after weighting were considered indicative of adequate balance. A covariate balance plot (Love plot) comparing pre- and post-weighting SMDs was performed. IPTW was applied using both stabilized and trimmed weights to control for baseline confounding while minimizing the influence of extreme propensity scores. Stabilized weights were used to reduce variance and improve estimate precision, whereas trimming excluded patients with extreme scores (below the 1 st and above the 99th percentile) to enhance model robustness. Univariate and multivariate logistic regression analyses were performed to assess the association between PET parameters and PSA50 or PSA90 responses, and the accuracy in the prediction of these two endpoints was determined with Receiver-Operating Characteristic (ROC) analysis. Kaplan–Meier curves weighted by IPTW were generated for the binarized PET parameters, and log-rank tests were used to compare survival distributions. Univariate and multivariate Cox proportional hazards models were used to evaluate associations between PET parameters, PSA relapse, and TTF. Model discrimination was assessed using Harrell’s C-index, and p values < 0.05 were considered statistically significant. All analyses were performed using Python version 3.13.

## Results

### Patient characteristics

**Fig. 1:**
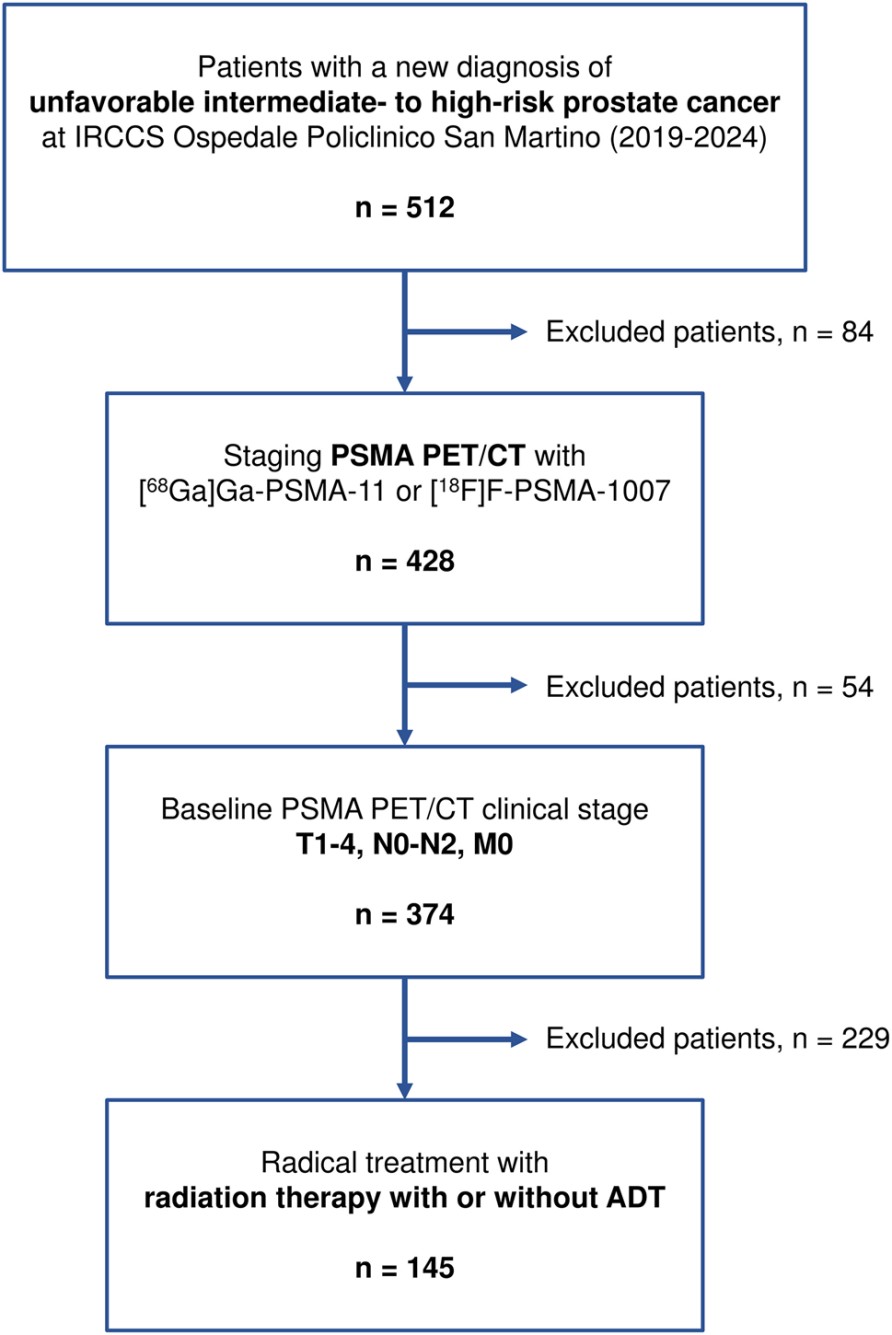
Flowchart of patients’ selection

The flowchart showing patients’ selection according to inclusion and exclusion criteria is represented in Fig. 1. A total of 145 patients with unfavorable intermediate to high-risk PCa were included in the study (Table 1). The median age at PSMA PET/CT was 76.0 years (IQR: 73.0–79.0). The median initial PSA was 8.9 ng/mL (IQR: 6.5–15.5). Most patients had an ISUP grade group of 3 (39.3%) or 2 (26.9%). The majority of patients were staged with [^18^F]F-PSMA-1007 (89.7%). Lymph-node involvement (cN +, including both miN1 and miN2) was present in 28.3% of cases. Median RT doses to prostate and pelvic lymphatics were 64.0 Gy (IQR: 64.0–64.0) and 46.0 Gy (IQR: 46.0–46.0), respectively, both delivered in a median number of 20 fractions (IQR: 20.0–20.0). Concurrent ADT was administered in all patients, and the most common duration was 12–24 months (60.7%). The median follow-up duration was 20.5 months (range 1–50 months).

**Table 1:** Clinical characteristics of enrolled patients

Characteristic	Value
**Patients, n**	**145**
Age (years)	
Median (IQR)	76.0 (73.0–79.0)
Initial PSA (ng/mL)	
Median (IQR)	8.9 (6.5–15.5)
≤ 10 ng/mL	79 (54.5%)
10–20 ng/mL	23 (15.9%)
> 20 ng/mL	43 (29.6%)
ISUP grade group, *n* (%)	
Grade 1	3 (2.1%)
Grade 2	39 (26.9%)
Grade 3	57 (39.3%)
Grade 4	38 (26.2%)
Grade 5	8 (5.5%)
PSMA-PET radiopharmaceutical	
[^18^F]F-PSMA-1007, n (%)	130 (89.7%)
Uptake time (minutes), median	95.0 (range 90.0–110.0)
[^68^ Ga]Ga-PSMA-11, n (%)	15 (10.3%)
Uptake time (minutes), median	64.0 (range 53.0–70.0)
Clinical stage at diagnosis, *n* (%)	
* T Stage*	
miT2	60 (41.4%)
miT3a	43 (29.6%)
miT3b	39 (26.9%)
miT4	3 (6.1%)
* N Stage*	
cN0	104 (71.7%)
cN +	41 (28.3%)
PSMA-PET parameters, median (IQR)	
SUVmax (Dominant lesion)	12.2 (8.8–20.6)
PSMA-TV (Total)	5.4 (3.0–13.0)
PSMA-TL (Total)	38.0 (21.6–77.5)
Radiation therapy, median (IQR)	
Dose to prostate (Gy)	64.0 (64.0–64.0)
Dose to pelvic lymphatics (Gy)	46.0 (46.0–46.0)
Fractions (number)	20.0 (20.0–20.0)
Concurrent ADT, *n* (%)	
< 12 months	32 (22.1%)
12–24 months	88 (60.7%)
> 24 months	25 (17.2%)
Treatment response	
PSA nadir after RT (ng/mL)	
Median (IQR)	0.01 (0.01–0.08)
PSA reduction (%)	
Median (IQR)	99.8 (99.0–99.9)
Outcome	
PSA relapse, n (%)	7 (4.8%)
Treatment failure, n (%)	13 (9.0%)
TTF (months), median	18.1 (1.1–49.8)

### Predictive value of PSMA-PET parameters

In univariate and multivariate logistic regression models, none of the PET parameters (SUVmax, PSMA-TL, PSMA-TV) were significantly associated with PSA50 or PSA90 responses. Odds ratios were close to 1 (see Supplementary Fig. 2), and area under the curve (AUC) values ranged from 0.49 to 0.55, indicating poor discriminatory power. Similarly, in Cox regression models for PSA relapse and TTF, no significant associations were found. However, PSMA-TV showed the numerically highest C-index (0.704 for PSA relapse, 0.674 for TTF), suggesting a potential prognostic role. Kaplan–Meier curves comparing patients with PET parameters above vs. below the median revealed no statistically significant differences in PSA relapse or TTF for any parameter (Supplementary Fig. 3 and Supplementary Fig. 4). However, the log-rank p value for PSMA-TV and TTF was 0.0599, approaching but not reaching significance.

**Fig. 2:**
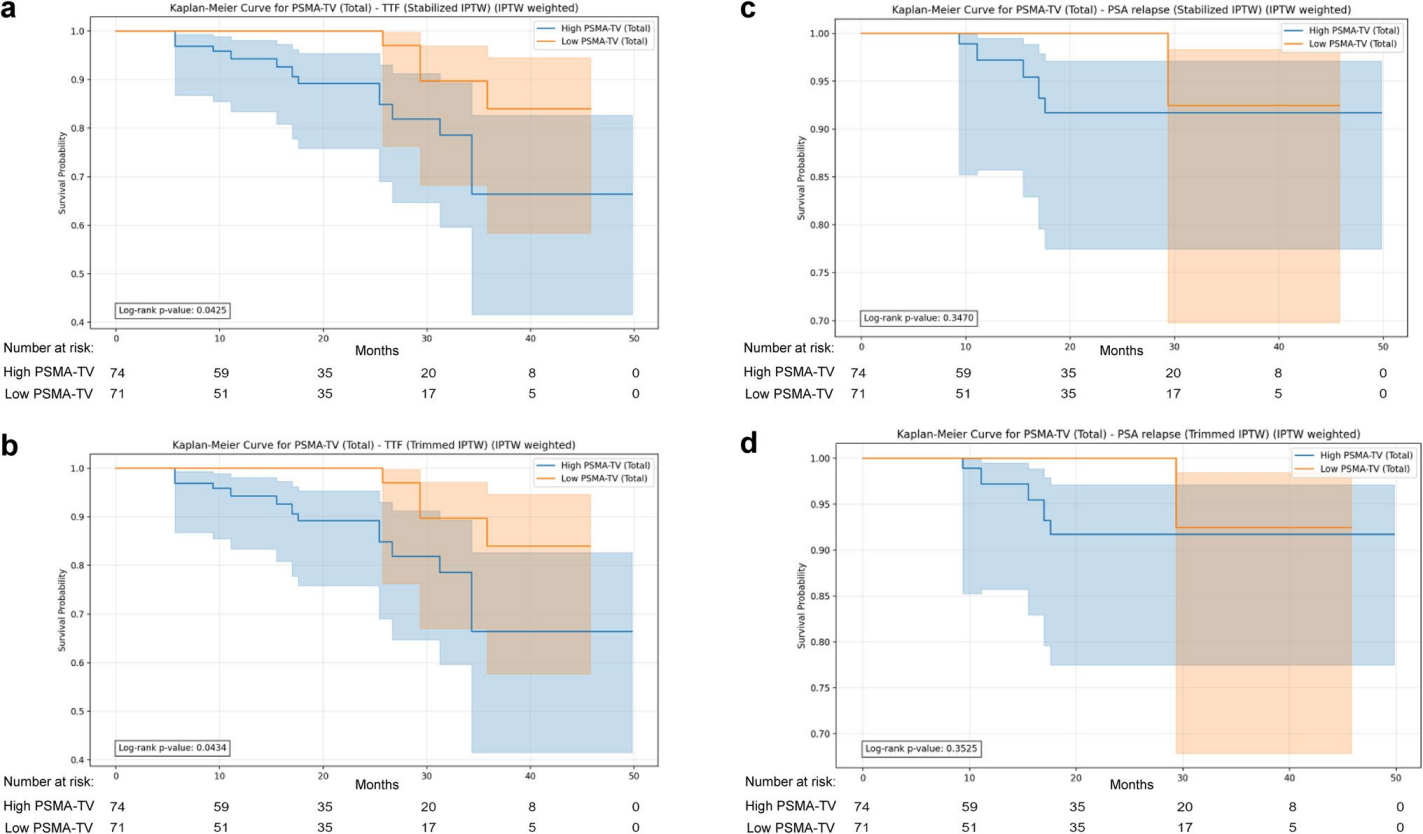
IPTW-weighted Kaplan–Meier curves for TTF (on the left) and PSA relapse (on the right), stratified by PSMA-TV (high vs. low, dichotomized at the median). IPTW was applied using stabilized (panels a-c) and trimmed weights (panels b-d) to adjust for baseline differences in initial PSA, ISUP grade, T stage, and N stage, while reducing the influence of extreme propensity scores

IPTW was applied to control for baseline differences based on initial PSA, ISUP grade, T stage, and N stage. In IPTW-weighted Kaplan–Meier analyses, PSMA-TV was significantly associated with TTF (*p* < 0.05) (Fig. 2a, b). Patients with high PSMA-TV had a shorter time to treatment failure. As shown in Supplementary Fig. 5, Kaplan–Meier curves performed grouping patients according to each individual counfounder used for IPTW calculation reached significance. Cox regression analysis confirmed this result (HR 3.79, 95% CI 1.04–13.81; *p* < 0.05) (Table 2). On the contrary, no significant differences were observed for PSMA-TV and PSA relapse (*p* > 0.3) (Fig. 2c, d).

**Table 2:** Cox regression analysis for PSMA-TV as a predictor of TTF

Variable	HR (95% CI)	*p value*
PSMA-TV (binary)		
Low PSMA-TV	1 (Ref.)	
High PSMA-TV	3.79 (1.04–13.81)	0.043
PSMA-TV (tertiles)		
First tertile	1 (Ref.)	
Second tertile	2.27 (0.41–12.40)	0.345
Third tertile	4.07 (0.84–19.70)	0.081
PSMA-TV (quartiles)		
First quartile	1 (Ref.)	
Second quartile	0.65 (0.06–7.15)	0.727
Third quartile	2.92 (0.57–15.09)	0.200
Fourth quartile	3.00 (0.58–12.79)	0.360

An exploratory analysis after grouping patients according to PSMA-TV tertiles and quartiles did not reach statistical significance in the prediction of either PSA relapse or TTF (Supplementary Figs. 6–7). These results were confirmed at Cox regression analysis (Table 2).

**Fig. 3:**
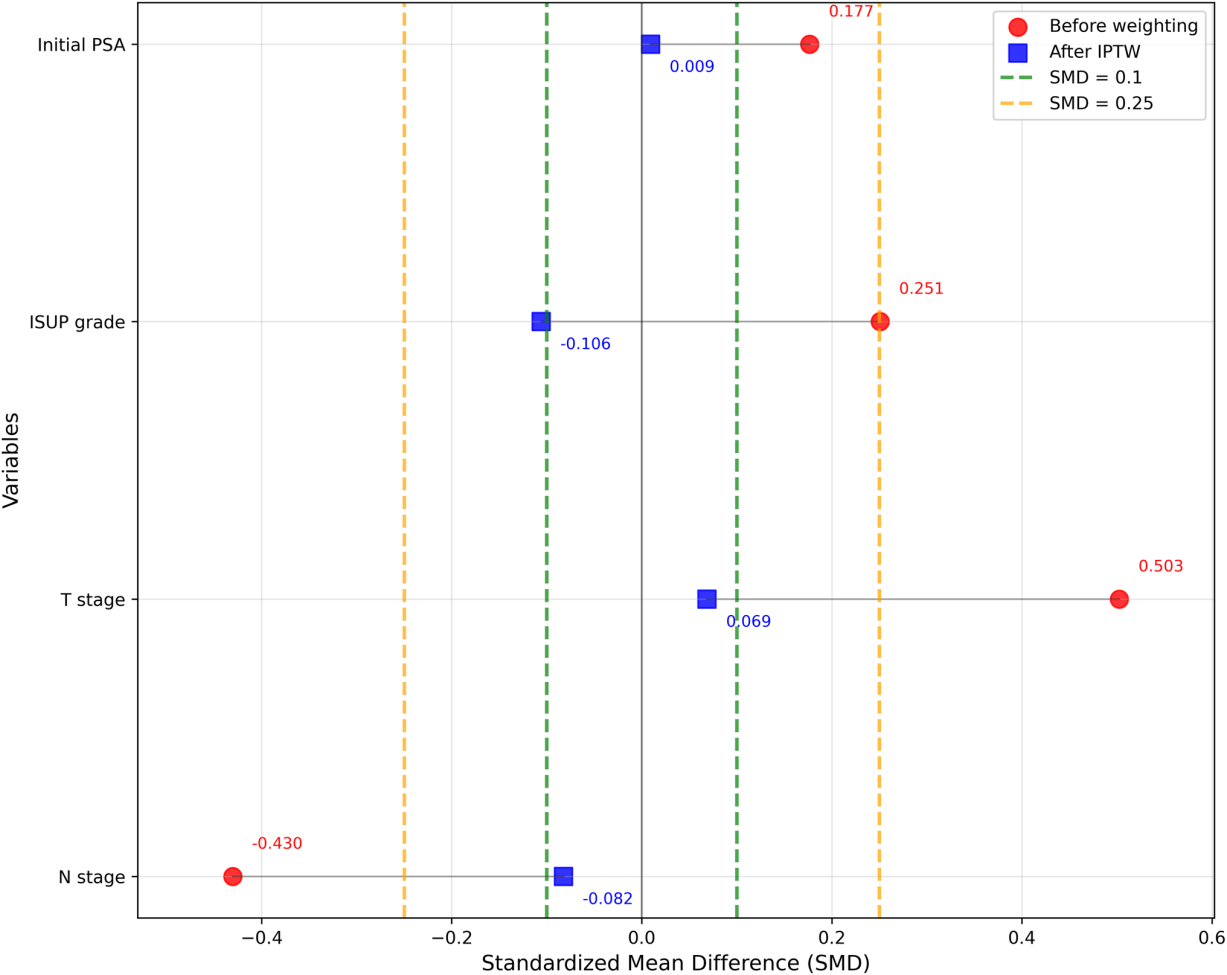
Love plot showing SMDs for prognostic variables included in the propensity score model before and after IPTW weighting in patients grouped according to median PSMA-TV

SMDs for each covariate included in the propensity score model (initial PSA, ISUP grade, T stage, and N stage), comparing high- and low-PSMA-TV groups before and after IPTW adjustment, are presented in Table 3 and Fig. 3. Following IPTW, all covariates achieved SMD showing an adequate balance between the two groups (Fig. 3).

**Table 3:** SMDs for prognostic variables included in the propensity score model before and after IPTW weighting in patients grouped according to median PSMA-TV

Variable	SMDs before IPTW weighting	SMDs after IPTW weighting
Initial PSA	0.177	0.009
ISUP grade	0.251	−0.106
T stage	0.503	0.069
N stage	−0.430	−0.082

Representative images of two patients presenting the same clinical stage at PSMA-PET performed for staging purposes, but with different total PSMA-TV (below and above the median value) are displayed in Fig. 3.

SUVmax and PSMA-TL were not significantly associated with either outcome (all *p* > 0.2).

These results suggest that PSMA-TV may be a significant prognostic factor for TTF when accounting for potential confounding variables through IPTW, but not for PSA relapse.

**Fig. 4:**
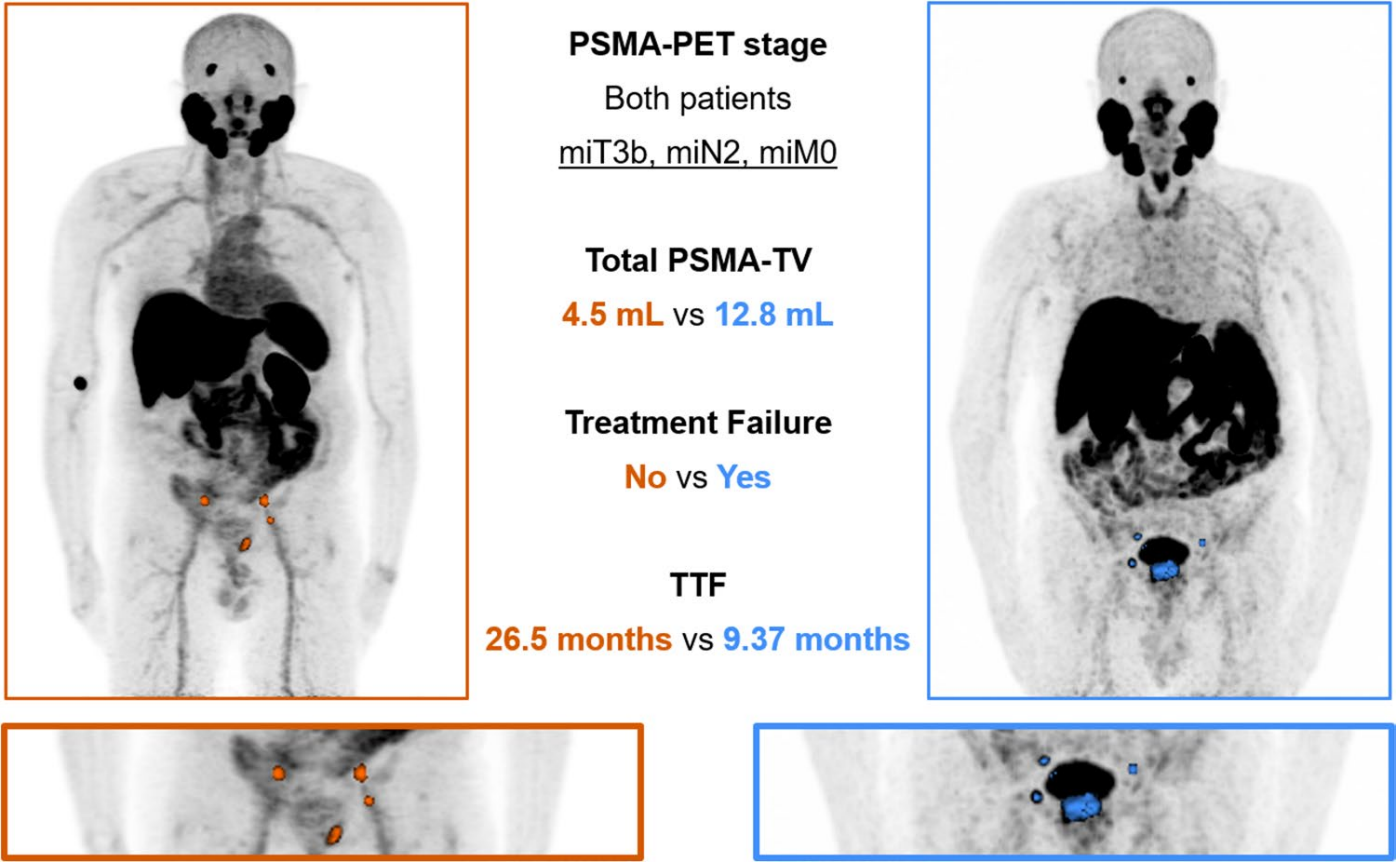
Maximum intensity projection images of PSMA-PET scans of two PCa patients submitted to initial staging before radical RT

## Discussion

In this retrospective study, we investigated the prognostic significance of PSMA PET/CT-derived quantitative parameters in patients with unfavorable intermediate to high-risk PCa undergoing curative-intent RT with ADT. Among the parameters analyzed, total PSMA-TV emerged as a statistically significant independent predictor of TTF. This result is in line with the previous findings highlighting the prognostic role of PSMA-TV in several clinical settings, while SUVmax and SUVmean (which is incorporated in the computation of PSMA-TL) showed a lower prognostic power [5, 6, 12]. Moreover, this association remained robust after IPTW, which adjusted for key clinical confounders including initial PSA, ISUP grade, and TNM staging. Notably, this association was not observed in unweighted analyses, reinforcing the importance of adjusting for baseline risk factors in observational cohorts [14]. Given the high efficacy of RT combined with ADT, PSA declines below the detection limit in nearly all patients. Consequently, PSA kinetics alone provide limited information for predicting treatment outcome. This highlights the need to investigate additional predictive parameters, such as PSMA-TV or other imaging-based biomarkers, to better stratify patients at risk of treatment failure.

The observed relationship between higher PSMA-TV and shorter TTF aligns with the existing literature demonstrating the value of PSMA PET/CT in the initial staging of PCa [2, 15–17], particularly in detecting nodal and distant metastases, surpassing conventional imaging modalities [4] and providing prognostic insights [5, 6]. Notably, in 16–48% of cases, the standard treatment planning might be modified according to PSMA PET/CT findings, due to the common identification of metastatic localizations outside the standard boundaries of pelvic lymph-node dissection or RT fields [18, 19]. However, the impact of these differences on the long-term outcome remains unclear.

In recent years, large multicenter initiatives, such as the CO-IMPACT consortium (https://www.coimpact-project.com/), have been launched to systematically evaluate how PSMA PET can inform RT in diverse clinical scenarios, including the initial staging of disease. A central hypothesis shared by these initiatives is that image-guided focal dose escalation targeting macroscopic PSMA-avid disease may improve oncological outcomes while minimizing toxicity to adjacent organs-at-risk.

Early studies have already demonstrated that the integration of PSMA PET/CT and/or multiparametric MRI (mpMRI) into RT workflows can significantly alter salvage treatment plans [20, 21]. However, while phase III clinical trials on focal dose escalation in localized PCa are ongoing, robust clinical evidence supporting dose escalation in nodal or low-volume metastatic settings—guided by advanced imaging—remains limited. The extent to which targeted intensification based on PSMA PET/CT or mpMRI influences long-term oncological outcomes or treatment-related toxicity has yet to be conclusively established.

The present study contributes to this evolving landscape by demonstrating that PSMA-TV is independently associated with time to treatment failure (Fig. 4), supporting its potential role as a risk-stratifying biomarker. This finding is in line with the well-established prognostic role of the metabolic tumor volume (MTV), a volumetric measure derived from [^18^F]-Fluorodeoxyglucose (FDG) PET, in other solid neoplasms [22, 23], often outperforming SUV-based indices for patients’ risk stratification.

From a clinical perspective, the identification of PSMA-TV as a significant prognostic biomarker opens the door to risk-adapted strategies in PCa management. In fact, our patients received a standard RT protocol, while new approaches that modulate the treatment according to imaging findings are emerging. Incorporating PSMA-TV into clinical workflows could facilitate individualized treatment strategies, including focal dose escalation guided by PSMA-PET, integration with systemic therapies in patients with imaging-based high risk of treatment failure, or tailored surveillance based on the individual risk of recurrence. This personalized approach may be especially relevant given the limitations of uniform dose escalation in the pelvic region, where critical structures such as the bladder and rectum constrain the ability to safely increase radiation doses to the entire prostate or nodal basin [24, 25]. In this context, accurate volumetric assessment of intra- and extraprostatic tumor burden via PSMA PET/CT may guide selective intensification. Prospective validation in multicenter cohorts and exploration of correlations with other endpoints, such as progression-free survival, time to systemic therapy, or castration resistance, are essential to confirm the generalizability of our findings and may further reinforce the application of PMSA-TV in clinical practice.

Several limitations of the present study should be considered. First, the retrospective design is inherently susceptible to confounding and selection bias, despite the application of IPTW. Second, the sample size may limit the power to detect smaller effect sizes, particularly in subgroup analyses. External validation using independent, prospective datasets with scanners’ harmonization is necessary before PSMA-TV can be incorporated into routine clinical workflows. Potential interactions between PSMA-TV and the duration of androgen deprivation therapy were not explored, as ADT duration was determined according to NCCN risk classification and not influenced by PSMA-PET findings. Furthermore, the limited number of events did not allow the calculation of specific PSMA-TV cut-offs for building a model with reliable performances. Similarly, we did not assess the added prognostic value of PET-derived parameters using formal methods such as net reclassification improvement or decision curve analysis, due to the limited sample size and number of events. Future studies with larger cohorts are warranted to explore this aspect. Standardization of PSMA-TV measurement [26], segmentation criteria accounting for different radiopharmaceuticals, or eventual integration with AI-based segmentation tools [27] may improve reproducibility in future studies. Finally, in the present study, due to the relatively small patient sample, we were unable to perform sensitivity analyses to assess potential differences in the predictive value of PSMA-TV depending on the specific PSMA-targeted radiopharmaceutical used. On one hand, this issue may be clinically relevant: in the oligorecurrent setting, where PET/CT is used to guide metastasis-directed therapy, several studies have shown that the choice of PET tracer can impact oncological outcomes [28–32]. Specifically, the use of [^68^ Ga]Ga-PSMA-11 versus [^18^F]F-PSMA-1007 has been associated with differing long-term prognoses [33, 34]. However, data from the PROMISE registry [5] indicate that PSMA-TV remains a significant prognosticator with similar hazard ratios across patient groups stratified by [^68^ Ga]Ga-PSMA-11 or [^18^F]-based PSMA tracers. Thus, although further studies are warranted to clarify these findings, it may be hypothesized that the prognostic value of PSMA-derived volumetric parameters such as PSMA-TV is independent of the tracer used.

## Conclusions

Our findings highlight PSMA-TV as a promising imaging-derived biomarker independently associated with time to treatment failure. While further prospective validation is needed, its integration into clinical workflows could support more personalized and risk-adapted RT strategies in PCa management.

## Supplementary Information

Below is the link to the electronic supplementary material.

Supplementary file1 (DOCX 21032 KB)

## Data Availability

M.B. is responsible for data integrity and accuracy of the analyses. Raw data are available upon specific requests.
