# Supplementary material for: Baseline PSMA tumor volume as a prognostic marker in radical radiotherapy for prostate cancer: a propensity score-weighted retrospective analysis
Source: Ann Nucl Med. 2025 Oct 10;40(2):178–87. doi: 10.1007/s12149-025-02118-4 (PMC12923420; doi:10.1007/s12149-025-02118-4)

*Annals in Nuclear Medicine*

**Baseline PSMA Tumor Volume as a prognostic marker in radical radiotherapy for prostate cancer: a propensity score-weighted retrospective analysis**

Francesco Lanfranchi^1^, Liliana Belgioia^2,3^, Daniele Vita^2^, Jacopo Passoni^2^, Sara Mastrogiovanni^2^, Alessandra Catanoso^2^, Luca Sofia^2^, Valentina Pau^4^, Stefano Raffa^4^, Silvia Chiola^4^, Maria Isabella Donegani^4^, Roberta Piva^4^, Mattia Riondato^4^, Michela Marcenaro^3^, Giorgia Timon^3^, Cecilia Marini^4,5^, Salvina Barra^3^, Gianmario Sambuceti^2,4^, Matteo Bauckneht^2,4^*

^1^ Department of Experimental Medicine (DIMES), University of Genova, Genova, Italy

^2^ Department of Health Sciences (DISSAL), University of Genova, Genova, Italy

^3^ Radiation Oncology Unit, IRCCS Ospedale Policlinico San Martino, Genova, Italy

^4^ Nuclear Medicine Unit, IRCCS Ospedale Policlinico San Martino, Genova, Italy

^5^ Institute of Bioimaging and Complex Biological Systems, National Research Council (CNR), Milano, Italy

***Correspondence:** Matteo Bauckneht, MD, PhD, Nuclear Medicine Unit, IRCCS Ospedale Policlinico San Martino and Department of Health Sciences (DISSAL), University of Genova, 16132 Genova, Italy; e-mail: [matteo.bauckneht@unige.it](mailto:matteo.bauckneht@unige.it)

**Supplementary Figure 1.** Representative sagittal, transaxial, and coronal fused PSMA PET/CT images showing the segmentation of the volume of interest into the prostate gland for the calculation of semiquantitative parameters.


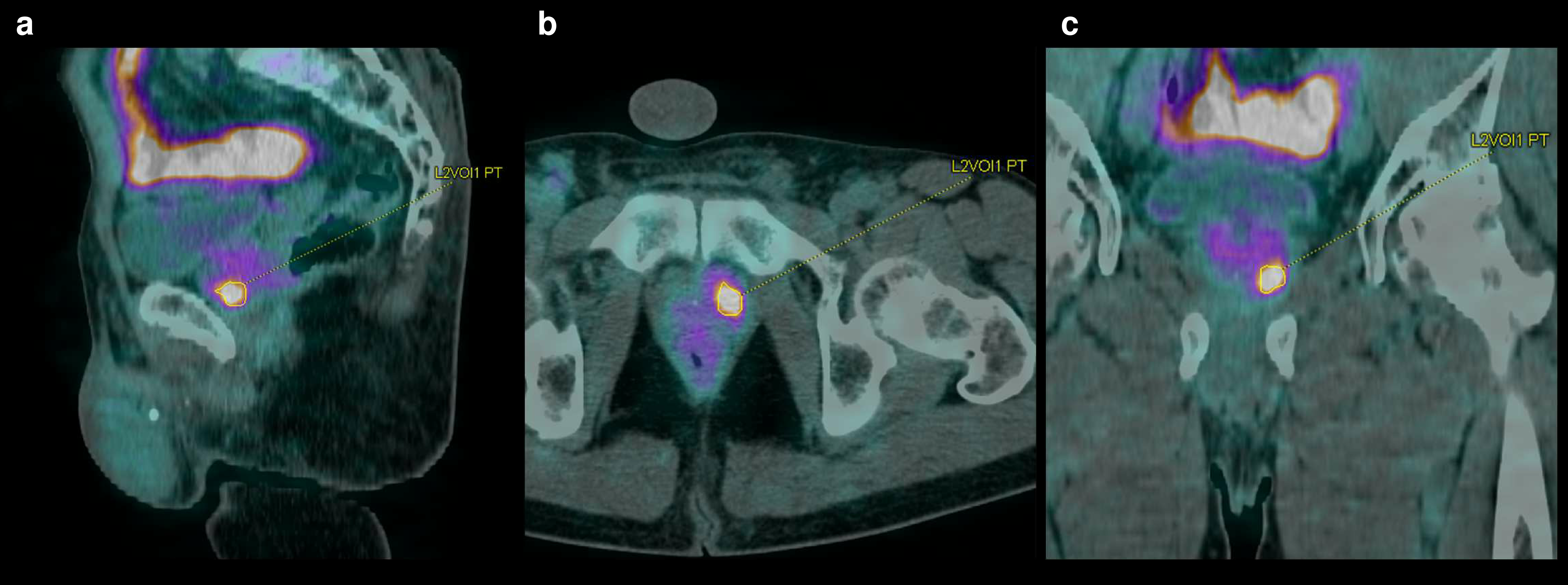


**Supplementary Figure 2.** Univariate analysis assessing the association between PSMA-PET parameters and outcomes.


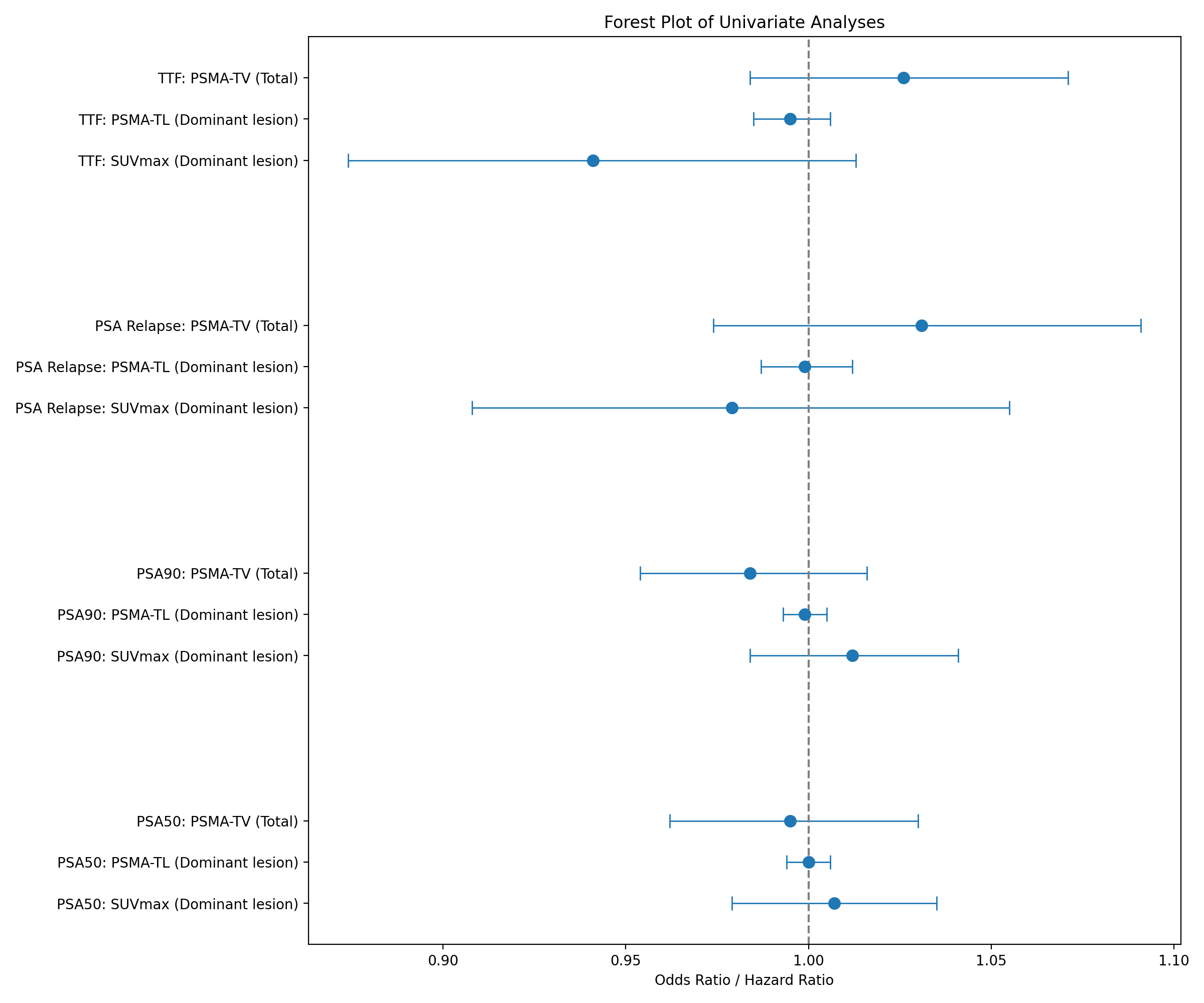


**Supplementary Figure 3.** Raw Kaplan–Meier curves for PSA relapse, stratified by PSMA PET-based measures (high vs. low, dichotomized at the median).

**PSA relapse**


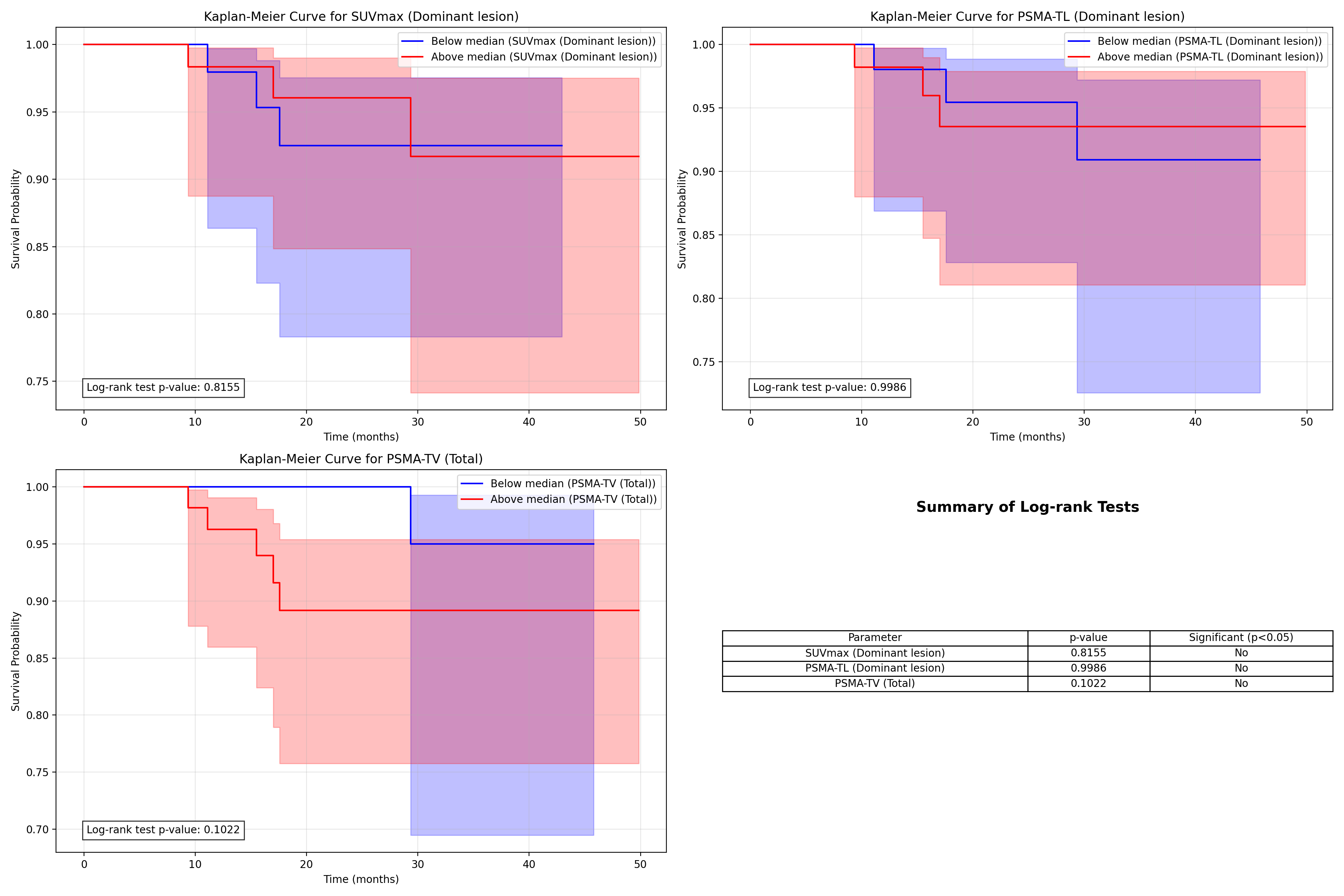


**Supplementary Figure 4.** Raw Kaplan–Meier curves for TTF, stratified by PSMA PET-based measures (high vs. low, dichotomized at the median).

**Time to treatment failure**


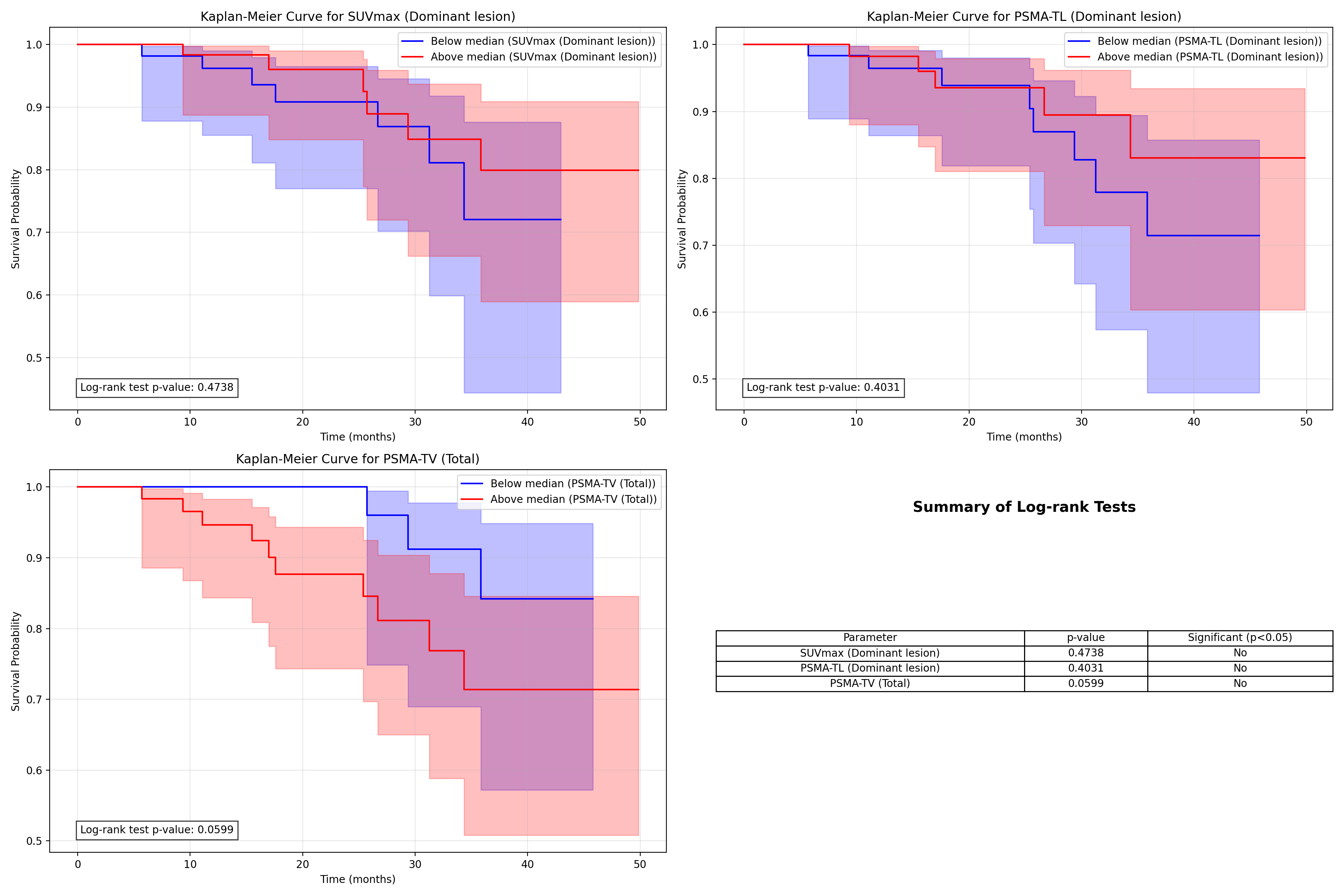


**Supplementary Figure 5.** Kaplan–Meier curves for TTF, stratified by initial PSA levels, ISUP grade, and PSMA-PET T and N stage.


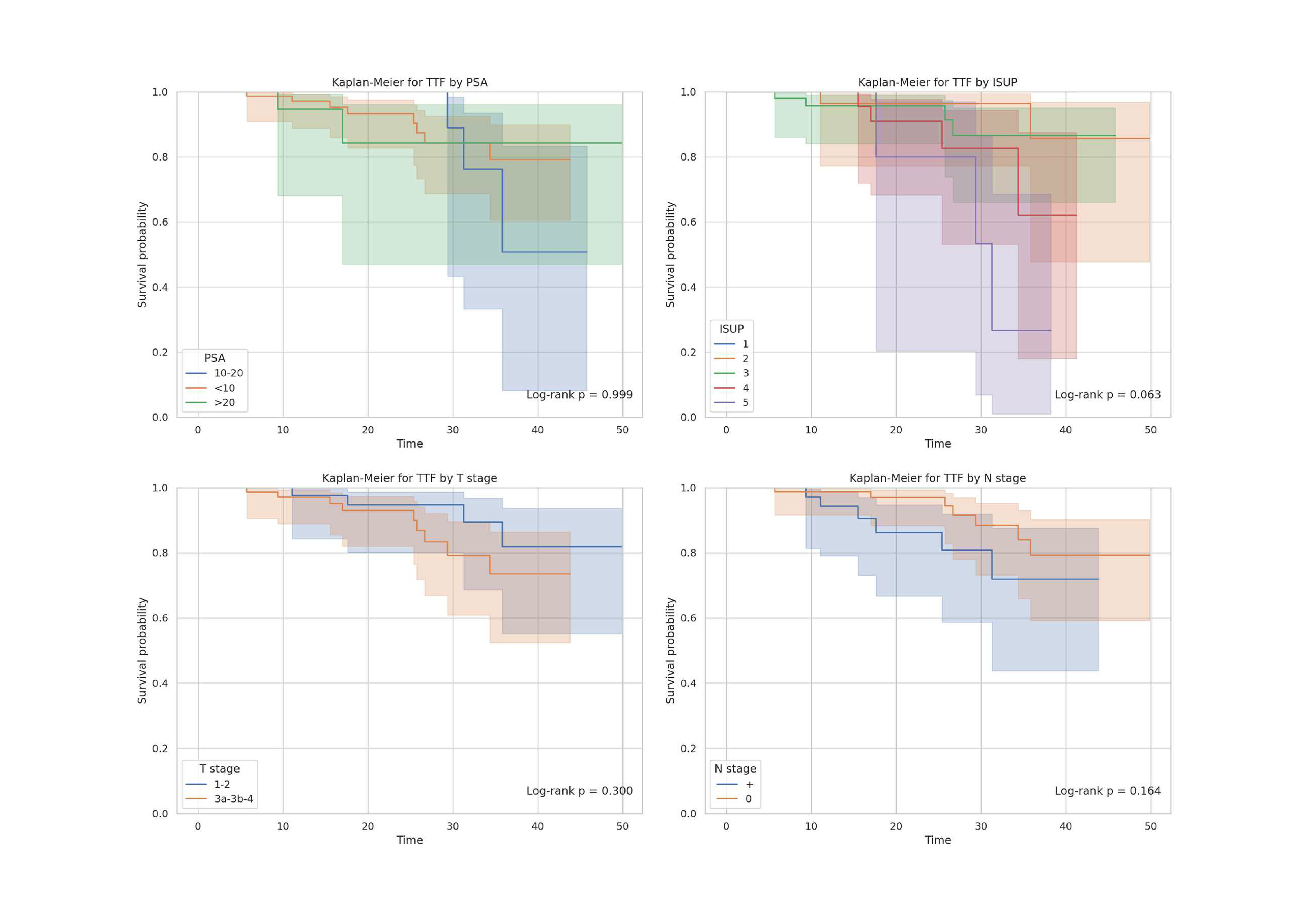


**Supplementary Figure 6.** IPTW-weighted Kaplan–Meier curves for TTF (on the left) and PSA relapse (on the right), stratified by PSMA-TV (tertiles).

**
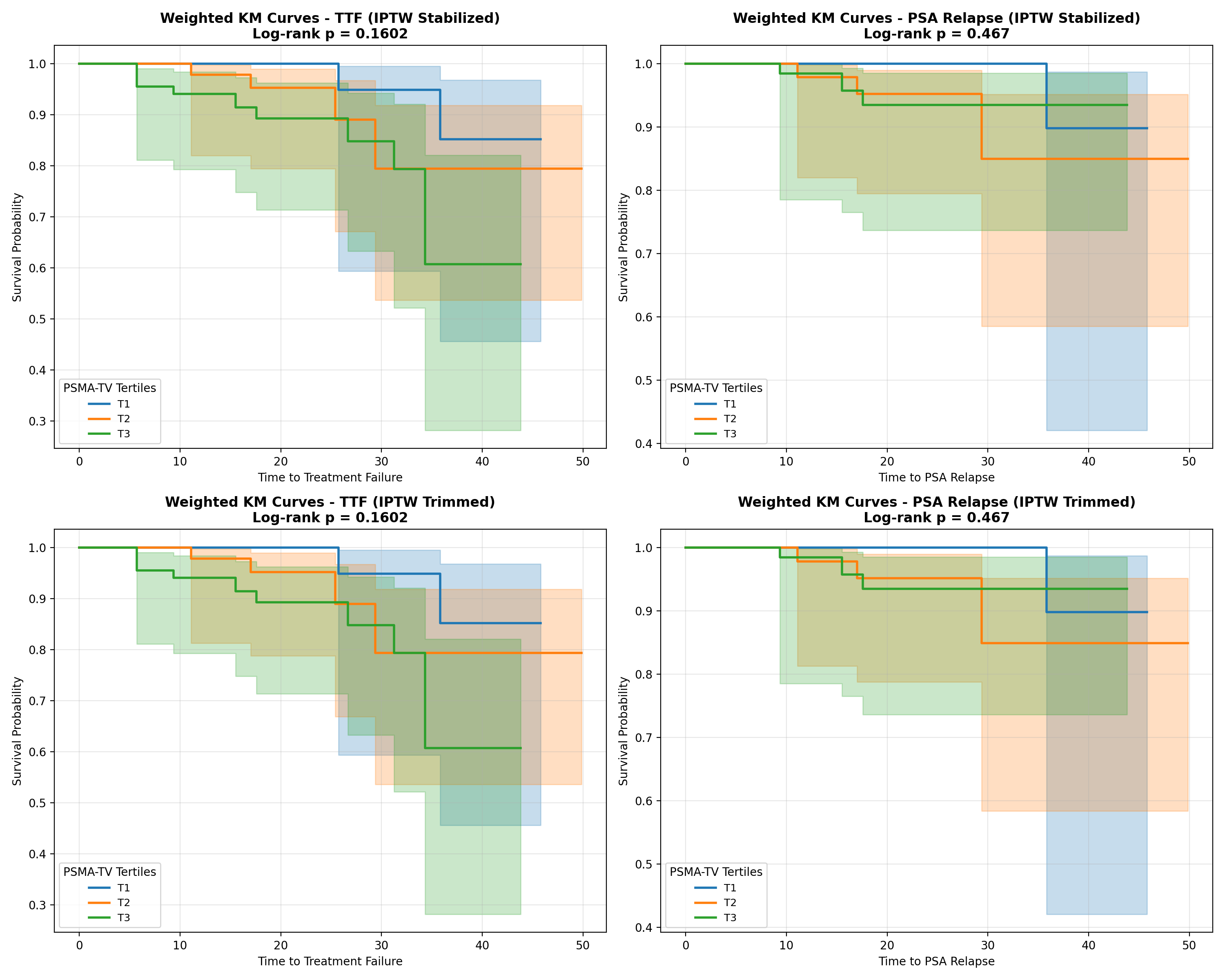
**

**Supplementary Figure 7.** IPTW-weighted Kaplan–Meier curves for TTF (on the left) and PSA relapse (on the right), stratified by PSMA-TV (quartiles).


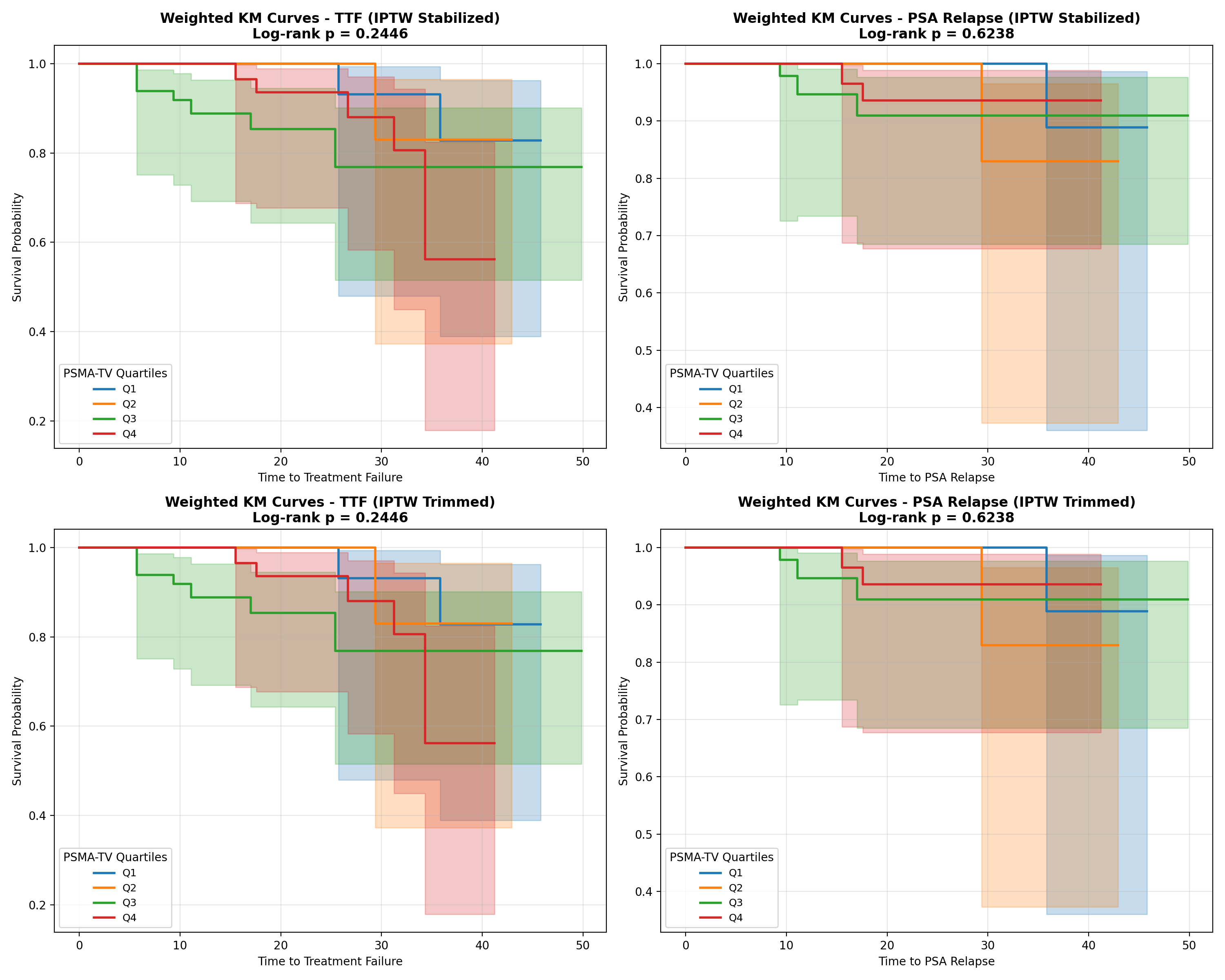

Supplement: Supplementary file 1 — Supplementary file1 (DOCX 21032 KB) [file 12149_2025_2118_MOESM1_ESM.docx]
